# Mechanistic insights into ADXS11-001 human papillomavirus-associated cancer immunotherapy

**DOI:** 10.1186/s40661-017-0046-9

**Published:** 2017-06-02

**Authors:** Brett A. Miles, Bradley J. Monk, Howard P. Safran

**Affiliations:** 1 Division of Head and Neck Cancer Surgery, Department of Otolaryngology, Icahn School of Medicine at Mount Sinai, One Gustave L. Levy Place, New York, NY 10029 USA; 2 Division of Gynecologic Oncology, University of Arizona College of Medicine, Creighton University School of Medicine at Dignity Health St. Joseph’s Hospital and Medical Center, Phoenix, AZ USA; 3 Brown University Oncology Research Group, Providence, RI USA

**Keywords:** AXAL, ADXS11-001, Axalimogene filolisbac, Cancer immunotherapy, Mechanism of action, Human papillomavirus, Vaccine therapy

## Abstract

Immune responses to the facultative intracellular bacterium *Listeria monocytogenes* (*Lm*) are robust and well characterized. Utilized for decades as a model of host-disease immunology, *Lm* is well suited for use as an immunotherapeutic bacterial vector for the delivery of foreign antigen. Genetic modification of *Lm* has been undertaken to create an attenuated organism that is deficient in its master transcriptional regulator, protein-related factor A, and incorporates a truncated, nonhemolytic version of the listeriolysin O (LLO) molecule to ensure its adjuvant properties while also preventing escape of the live organism from the phagolysosome. Delivery of a vaccine construct (*Lm*-LLO-E7; axalimogene filolisbac [AXAL] or ADXS11-001) in which the modified LLO molecule is fused with the E7 oncoprotein of human papillomavirus type 16 (HPV-16) consistently stimulates strong innate and E7 antigen-specific adaptive immune responses, resulting in reduction of tumor burden in animal cancer models. In the clinical setting, AXAL has shown early promise in phase I/II trials of women with cervical cancer, and several more trials are currently underway to assess the efficacy and safety of this antitumor vaccine in patients with HPV-positive head and neck and anal cancers.

## Introduction

### Human papillomavirus: Prevalence, molecular structure, and biology

Several infectious agents are considered to be necessary causal agents of human cancers. Among these, persistent infections involving the human papillomaviruses (HPV) are estimated to be responsible for 5.2% of all cancers worldwide, with the majority of cases occurring in developing countries [1]. Infection with high-risk, oncogenic HPV subtypes is directly attributable to all cases of cervical cancer, approximately 90% of anal cancers, approximately 40% of penile, vulvar, and vaginal cancers, and around 12% of head and neck cancers, mainly of the oropharynx [1]. HPV subtypes 16, 18, 31, and 45 are the most frequently encountered high-risk HPV types; subtypes 16 and 18 alone are the causative agents of more than 70% of cervical cancer cases [2].

HPV is a circular, double-stranded, non-enveloped, icosahedral DNA virus. The HPV genome contains six or seven early genes, denoted E1, E2, E4, E5, E6, E7, and E8, which are required for maintenance of the viral genome, DNA replication, regulation of transcription, stimulation of cell growth, and inhibition of tumor suppressor genes [3]. E6 and E7 are the major oncogenes of HPV, and are used by the virus to evade the host immune system and access cell replication machinery [4]. In addition, the HPV genome contains two late genes, L1 and L2, which encode the major and minor capsid proteins, respectively [3].

When the integrity of the host cutaneous or mucosal epithelium has been compromised (e.g., microabrasions or other trauma), HPV infects the basal epithelial cells and establishes an episome. As the infected cells differentiate, early and late viral proteins are expressed, leading to viral assembly and eventual viral shed. In high-risk, oncogenic HPV subtypes, the E6 protein targets the p53 tumor suppressor protein, whereas E7 binds to the active form of the retinoblastoma protein, thereby disrupting normal cell cycle regulation and providing the means to cause cellular alterations that potentially lead to neoplasia [3]. Cancer develops after a long latency period in which viral DNA persists, with ongoing viral integration into the host cell DNA, and continuous overexpression of the E6 and E7 early proteins, with consequent aberrant proliferation of the host cells [4, 5].

## Review

### *Listeria monocytogenes*: Versatile delivery vector for immunotherapy

*Listeria monocytogenes* (*Lm*) is an anaerobic, Gram-positive, facultative intracellular bacterium that is associated with opportunistic foodborne disease in susceptible hosts [6]. During active infection by *Lm*, the organism may disseminate via the bloodstream from the principal site of infection in the gastrointestinal tract and invade organs such as the spleen and liver, where it is phagocytosed by splenic and hepatic macrophages [7]. Following cellular invasion, *Lm* escapes the phagosome by secreting the pore-forming toxin listeriolysin O (LLO), a virulence factor that destroys the phagosomal membrane, and which allows the organism to undergo rapid cytosolic growth and actin nucleator A (ActA)-dependent cell-to-cell spread [8]. The entire *Lm* life cycle is dependent on the virulence gene and master transcriptional regulator protein-related factor A (prfA). ActA, an abundant surface protein that is upregulated more than 200-fold during intracellular growth in order for the bacterium to move toward the cell surface and spread to other cells [9], is activated in the host cytosol following allosteric activation of prfA, and subsequently mediates host actin polymerization. Once at the cell membrane, *Lm* forms a protrusion that is subsequently internalized by an adjacent macrophage, thereby disseminating the infection. Appropriate regulation of LLO and ActA by prfA is critical for *Lm* pathogenesis [8].

**Fig. 1:**
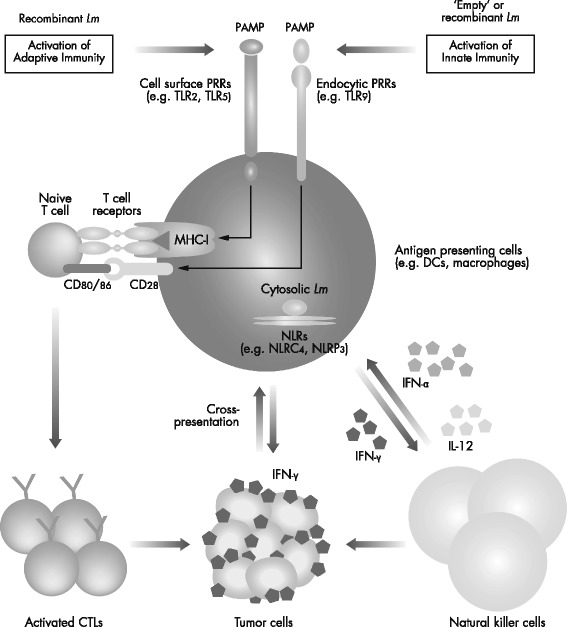
Innate and adaptive immunity mediated by *Lm*. Adapted from Promises and challenges for the development of Listeria monocytogenes-based immunotherapies. Brockstedt DG, Dubensky TW, 2008 [10]. Expert Review of Vaccines. 2008. Taylor & Francis Ltd. Reprinted by permission of Taylor & Francis Ltd. CD, cluster of differentiation; CTL, cytotoxic T lymphocyte; DCs, dendritic cells; IFN, interferon; IL-12, interleukin-12; *Lm*, *Listeria monocytogenes*; MHC-I, major histocompatibility complex class I; NLRs, nucleotide-binding oligomerization domain-like receptors; PAMP, pathogen-associated molecular pattern; PRRs, pattern recognition receptors; TLR, Toll-like receptor

*Lm* has the ability to activate both the innate and adaptive immune responses (Fig. 1) [7, 10]. Following infection with *Lm*, innate immune responses are rapidly triggered in a stepwise manner, with the hallmarks of early resistance to infection being the production of interferon-gamma (IFN-γ) by natural killer cells and the subsequent activation of macrophages. At the cell surface, Toll-like receptors (TLRs) are an important link between the pathogen and subsequent immune activation, with TLR2 and TLR5 involved in the recognition of *Lm* pathogen-associated molecular patterns, such as peptidoglycan, lipoteichoic acid, lipoproteins, and bacterial flagellins [7, 11]. Myeloid differentiation primary response protein 88 is important in the innate immune defense against *Lm*, where its role in transmitting TLR-mediated signals is a required element for the full activation of immune responses [12]. Whereas TLRs are extracellular pattern recognition receptors involved in the activation of the inflammasome and production of pro-inflammatory cytokines, the nucleotide-binding oligomerization domain-like receptors (NLRs) are involved in the detection of cytosolic pathogens [13]. In particular, NLRC4 and NLRP3 detect cytosolic *Lm* with consequent activation of the inflammasome, while a further NLR, AIM2, specifically senses the bacterial DNA of *Lm*. The ensuing inflammatory response ensures the infiltration of large numbers of neutrophils and then macrophages to the site of infection, where they help to limit bacterial growth and, in the case of macrophages, drive the subsequent adaptive immune response [14].

During phagocytosis by infiltrating macrophages, any *Lm* bacteria that have not escaped the phagosome are phagocytosed and their processed antigen fragments are presented on the cell surface via major histocompatibility complex (MHC) class II. This interaction between the bacterial peptide/MHC class II complex and T cells that are able to recognize the antigen via their own receptors subsequently leads to the activation of cluster of differentiation 4-positive (CD4+) T cells [14]. In addition, bacteria that have escaped the phagosome into the cytosol may release antigenic fragments that are presented by MHC class I molecules to CD8+ cytotoxic T cells, with both CD4+ and CD8+ T cells involved in final clearance of the infection and generation of protective immunity [14, 15]. *Lm* is a strong stimulator of CD8+ T-cell responses in particular, with CD8+ T cells undergoing rapid programming to become long-lived CD8+ memory T cells, which provide protection against subsequent *Lm* infections [16]. Dendritic cells are an important link between the innate and adaptive immune responses, with their activation in response to the TLR signaling cascade required for co-stimulation of T cells and the effective activation of cell-mediated immunity [14, 16]. The CD8α subset of conventional dendritic cells is most effective in supporting this CD8+ T-cell memory formation [15].

Because of its well-established and robust immunologic effects, as well as decades-long use as a model of host-disease immunology, strains of *Lm* have been deployed as a therapeutic bacterial vector for the delivery of foreign antigens in both the preclinical and clinical settings [15]. The utility of the *Lm* vector is achieved through its genetic recombination with a truncated, nonhemolytic form of LLO, which eliminates the cytolytic activity of *Lm* and associated cell toxicity while preserving the significant immunogenic and adjuvant properties of the organism. For example, ADXS31-164 is an *Lm*-based vaccine that expresses a chimeric human HER2/neu gene fused to a nonhemolytic LLO fragment, which is expressed in the highly attenuated *Lm* vector LmddA. The vector lacks antibiotic selection markers and has the ability to spread from cell to cell. Despite this level of attenuation, ADXS31-164 was able to disrupt immune tolerance toward the HER2/neu self-antigen, eliciting strong T-cell responses in experimental animal tumor models that resulted in a reduction in regulatory T cells (Tregs), an increase in the CD8+/Treg ratio, and a reduction in tumor growth [17].

In the preclinical setting, *Lm*-based vaccine strategies were shown to potentiate CD8+ T-cell responses and inhibit neoangiogenesis in mouse models of breast, cervical, and head and neck cancers [17–22]. Singh et al. [18] demonstrated that five unique HER2/neu fragments secreted as a fusion protein with a truncated, nonhemolytic form of LLO and expressed in recombinant *Lm* controlled the growth of established NT2 mammary tumors, with the antitumor effect driven by a population of anti-HER2/neu CD8+ cytotoxic T cells [18]. In a syngeneic 4 T1 mouse tumor model, vaccination with a melanoma-associated antigen b-*Lm*-LLO–based vaccine significantly reduced the number of metastases by 96% when compared to saline, and by 88% when compared to the vector control group (i.e., *Lm*-LLO alone) [19]. Administration of a vascular endothelial growth factor-targeted recombinant *Lm*-LLO–based vaccine in a mouse model of breast cancer led to eradication of some of the established tumors, reduction of microvascular density in the remaining tumors, and protection against tumor rechallenge and experimental metastases [20]. In an autochthonous mouse model for human epidermal growth factor receptor 2 (HER2)/neu + breast cancer, a novel human HER2/neu chimera *Lm*-based vaccine combining selected portions of individual fragments of the HER2/neu protein that contained most of the human leukocyte antigen epitopes prevented spontaneous tumor outgrowth, induced tumor regression in transplantable models, and prevented seeding of experimental lung metastases [21]. In a mouse model of HER2/neu-driven breast cancer, the *Lm*-LLO-CD105A and *Lm*-LLO-CD105B *Lm* recombinant vaccines that target endoglin (CD105) expressed in tumor vasculature were able to prevent neovascularization, thereby leading to therapeutic responses against primary and metastatic tumors [22].

In addition to breast tumor models, the antitumor activity of *Lm*-based vaccines has also been demonstrated in preclinical models of cervical and head and neck cancers [23–25]. A recombinant *Lm* construct that encoded the HPV-16 E7 gene was used to evaluate the potential potency of recombinant *Lm*-E7 as a therapeutic vaccine for cervical cancer in a syngeneic mouse model. When orally administered, the *Lm*-based vaccine induced an E7-specific cytotoxic T-cell response that could prevent and eradicate tumor growth in vaccinated mice because of enhancement of antigen-specific T-cell immunity [23]. These effects were confirmed by another study, which reported that an *Lm*-based HPV-16 E7 vaccine limited autochthonous tumor growth in a transgenic mouse model of HPV-16–transformed tumors [25]. In a mouse model of head and neck cancer, the administration of an *Lm*-based ActA vaccine expressing the E7 protein of HPV-16 caused complete regression of HPV+ tumors in six of eight tested mice [24]. Consistent with other tumor models, the antitumor response was driven by the activation of cytotoxic T cells.

### Listeriolysin O: Potent adjuvant for immunotherapy

**Fig. 2:**
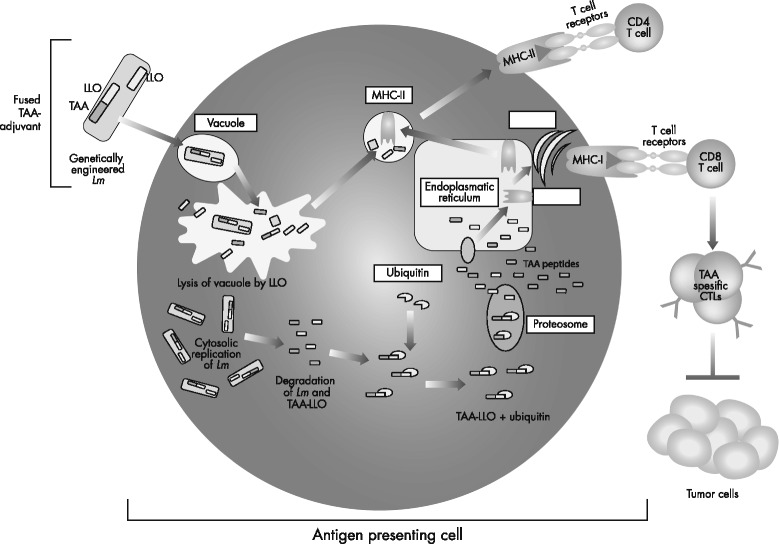
Schematic depiction of processing and presentation of the LLO-Ag fusion protein in an antigen-presenting cell. Adapted from Wallecha A, French C, Petit R, Singh R, Amin A, Rothman J. Lm-LLO-based immunotherapies and HPV-associated disease. J Oncol. 2012;2012:542851 [27], under Creative Commons Attribution 3.0 Unported (CC BY 3.0) license (https://creativecommons.org/licenses/by/3.0/). Figure is a derivative of the original. Ag, antigen; CD, cluster of differentiation; CTL, cytotoxic T lymphocyte; LLO, listeriolysin O; *Lm*, *Listeria monocytogenes*; MHC-I, major histocompatibility complex class I; MHC-II, major histocompatibility complex class II; TAA, tumor-associated antigen

LLO is a 529-amino acid hemolytic pore-forming protein crucial for the intracellular escape of *Lm* from the phagolysosome of infected cells [26]. In the context of tumor immunology, LLO is a very useful adjuvant because of its immunologic properties. Fusion of tumor antigens to the first 420-amino acid sequence of LLO, which excludes the hemolytic domain, helps to facilitate secretion of the antigen, increase antigen presentation, and stimulate maturation of dendritic cells (Fig. 2) [25, 27]. Details of this bioengineered version of the LLO molecule were first published by Gunn et al., who prepared two recombinant *Lm* strains, one expressing the E7 protein of HPV-16 with no attempt to modify the LLO molecule (*Lm*-E7), and the second expressing E7 as a fusion protein joined to nonhemolytic LLO (*Lm*-LLO-E7) [28]. The two strains induced qualitatively different T-cell immune responses that correlated with their ability to induce regression of established HPV+ tumors in mice. *Lm*-LLO-E7, but not *Lm*-E7, induced the regression of E7-expressing tumors in a syngeneic mouse model with tumor regression dependent on a CD8+ T-cell response. The antitumor response to *Lm*-LLO-E7, but not *Lm*-E7, was reduced considerably with the depletion of CD4+ T cells, indicating the potency of the nonhemolytic LLO molecule as an immunologic adjuvant compared to the native LLO molecule. In contrast, *Lm*-E7 was shown to be an effective tumor immunotherapy in mice depleted of CD4+ T cells. Furthermore, antibody-mediated depletion of CD25+ cells improved the efficacy of *Lm*-E7 treatment [28]. In the years since, preclinical studies have shown that *Lm*-LLO-E7 is able to stimulate the expression of a wide range of pro-inflammatory cytokines by dendritic cells, such as interleukin-2 (IL-2), IL-12, tumor necrosis factor-α, and IFN-γ, as well as promote dendritic cell maturation, activate CD4+ T-cell–mediated adaptive immune responses, induce tumor antigen-specific CD8+ cytotoxic T cells, break immunologic tolerance, maintain protective immunity, and block tumor reoccurrence [29, 30]. Additionally, LLO is capable of inducing chemokines and co-stimulatory molecules crucial for the development of potent innate and adaptive immune responses.

### Axalimogene filolisbac (ADXS-HPV)

#### Molecular mechanism of action and immunotherapeutic effects

**Fig. 3:**
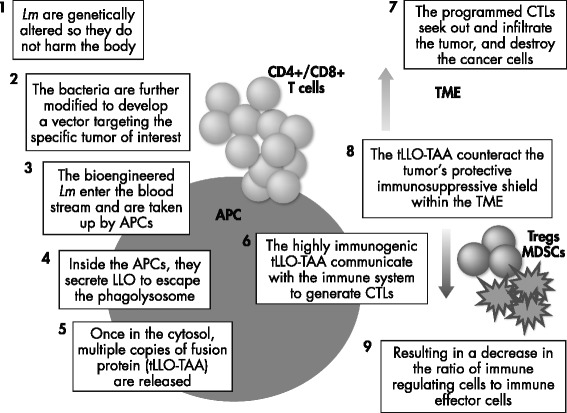
Step-by-step *Lm*-LLO immunomodulation. APCs, antigen-presenting cells; CD, cluster of differentiation; CTL, cytotoxic T lymphocyte; MDSC, myeloid-derived suppressor cell; TAA, tumor-associated antigen; tLLO, truncated LLO; TME, tumor microenvironment; Treg, regulatory T cell

Axalimogene filolisbac (AXAL, or ADXS11-001) is a live, irreversibly attenuated *Lm*-LLO-E7 immunotherapy specifically developed for the treatment of HPV-associated cancers [31] (Fig. 3). As in earlier editions of *Lm*-LLO-E7, AXAL secretes an antigen-adjuvant fusion protein consisting of a truncated, nonhemolytic fragment of LLO fused to HPV-16 E7. AXAL was bioengineered from the prfA-deficient XFL-7 *Lm* strain, which renders the organism nonvirulent and also unable to escape the phagolysosome of the infected cell [32]. The strain was transformed using the pGG55 multicopy plasmid, which contains an expression cassette with the E7 gene fused to a truncated *hly* gene that encodes the first 441 amino acid residues of LLO and additionally contains a mutated copy of the prfA gene to partially restore XFL-7 virulence needed for plasmid retention in vivo.

AXAL targets tumors through a mechanism of action that results in activation of innate and adaptive immune responses. Briefly, the attenuated *Lm* expressing the HPV antigen fused to LLO is taken up by antigen-presenting cells via phagocytosis [33, 34]. Through its induction of pro-inflammatory cytokines from natural killer cells, recruitment of monocytes from the peripheral blood to the site of inflammation, and maturation of local dendritic cells, LLO helps to mediate a number of immunostimulatory effects that are an essential bridge between the innate and adaptive immune responses [33]. Antigenic peptides that result from the phagocytosis and breakdown of *Lm* are presented via MHC class II to antigen-specific CD4+ T cells. The immunogenic nature of LLO is further associated with a peptide sequence rich in proline, glutamic acid, serine, and threonine, which likely targets the protein for rapid ubiquitin-mediated proteasomal degradation, with antigenic fragments processed via this cytosolic pathway and subsequently presented via MHC class I to antigen-specific CD8+ T cells. Thus, both arms of the adaptive immune system are stimulated, resulting in the generation of strong T-cell–mediated effector immune responses and protective immunity [33, 34].

#### AXAL responses in mouse tumor models

Because of its capacity to effectively stimulate innate immunity and both arms of the adaptive immune response, AXAL presents attractive immunotherapeutic effects, which have been reported in both preclinical and clinical (Table 1 [25, 28, 32, 35–49]) studies. A study by Hussain and Paterson [35] shed light on the findings of Gunn et al. [28], who showed that antibody-mediated depletion of CD25+ cells improved the antitumor efficacy of *Lm*-E7 in a mouse cancer model. Hussain and Paterson showed in tumor-bearing mice that CD4 + CD25+ Tregs secreting transforming growth factor-β and the anti-inflammatory cytokine IL-10 are preferentially induced in mice vaccinated with *Lm*-E7, emphasizing the complexity of *Lm*-based immunotherapy. In a separate study, Peng et al. [36] reported that the ability of *Lm*-E7 and *Lm*-LLO-E7 vaccines to induce an antitumor response is correlated with myeloid dendritic cell maturation, as only *Lm*-LLO-E7 was able to induce IL-2 production by dendritic cells while also stimulating significantly higher levels of MHC class II molecules and co-stimulatory molecules necessary for stimulation of naive T cells [36]. This effect was independent of the E7 antigen, again indicating the adjuvant properties of LLO.

**Table 1:**
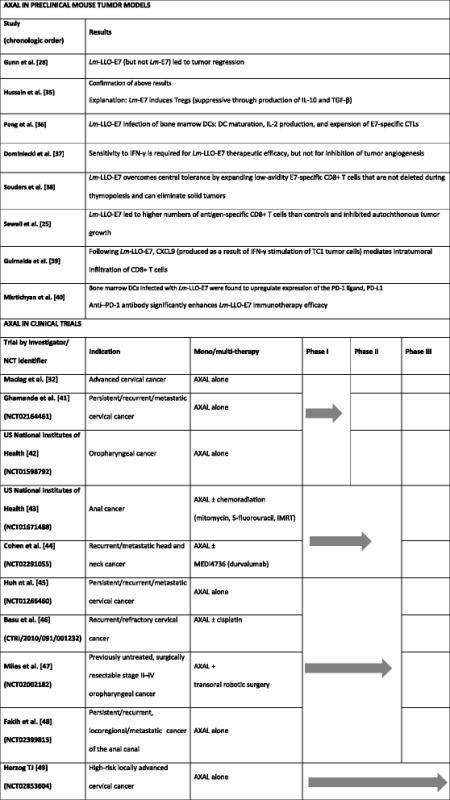
Overview of AXAL in preclinical and clinical studies

Abbreviations: *CTRI* Clinical Trials Registry – India, *DC* dendritic cell, *IMRT* intensity-modulated radiation therapy, *NCT* National Clinical Trial

Loss of responsiveness to IFN-γ provides an immune escape mechanism for many human tumors, yet tumor sensitivity to IFN-γ was not required for inhibition of tumor angiogenesis or infiltration of CD4+ and CD8+ T cells to the tumor site in response to *Lm*-LLO-E7 in preclinical models [37]. Dominiecki et al. used the TC1 tumor cell line, which is immortalized with HPV E6 and E7 proteins and rendered unresponsive to IFN-γ. Although *Lm*-LLO-E7 was unable to induce tumor regression in the IFN-γ–insensitive model possibly because of an inability of the infiltrating T cells to penetrate the tumor mass, the capability of *Lm*-LLO-E7 to inhibit tumor angiogenesis in this model is nevertheless an encouraging finding. Using a similar model, a more recent preclinical study reported that administration of *Lm*-LLO-E7 increases the secretion of chemokine (C-X-C motif) ligand 9 (CXCL9) by TC1 tumor cells and mediated the intratumoral infiltration of CD8+ T cells [39]. This effect was IFN-γ dependent, since anti–IFN-γ antibody treatment resulted in a reduction in CXCL9 expression and a resultant decrease in the proportion of CD8+ T cells. In a transgenic mouse model of HPV-transformed cancer, *Lm*-LLO-E7 was shown to overcome tumor-induced central tolerance by expanding low-avidity and low-frequency E7-specific CD8+ T cells, which eradicate E7-expressing thyroid mouse tumors [38]. In an effort to evaluate the systemic immunologic effects that differentiate *Lm*-LLO-E7 vaccination from its control vector lacking E7 protein expression, Sewell et al. [25] showed that mice treated with *Lm*-LLO-E7 had significantly smaller tumors than control mice and possessed higher numbers of antigen-specific CD8+ T cells in the spleens, tumors, and peripheral blood [25].

Another tumor immune escape mechanism and therefore barrier for successful immunotherapy is tumor-mediated inhibitory responses that are effected via programmed cell death protein 1 (PD-1) interactions with its ligands, PD-L1 and PD-L2. A recent study conducted in a TC1 mouse tumor model showed that the combination of *Lm*-LLO-E7 with an anti–PD-1 antibody that blocks the PD-1/PD-L1 interaction significantly improved the immunotherapeutic efficacy of treatment compared with *Lm*-LLO-E7 alone [40]. In particular, the combination treatment led to a significant reduction in Tregs and myeloid-derived suppressor cells (MDSCs) in the spleen and tumor microenvironment, and significantly enhanced antigen-specific CD8+ T-cell peripheral and tumoral immune responses, thereby prolonging survival and promoting the complete regression of tumors in mice.

#### Brief overview of AXAL in clinical studies

Following the positive results obtained in the preclinical setting, assessment of the efficacy and safety of AXAL immunotherapy was initiated in phase I/II clinical trials conducted in patients with HPV-associated cancers, including cervical cancer, head and neck cancer, and anal cancer (Table 1 [25, 28, 32, 35–49]).

In patients with cervical cancer, AXAL was assessed in several phase I/II trials either as monotherapy or in combination with other anticancer therapies. The safety of AXAL was first assessed in 2009 in a phase I trial in 15 patients with previously treated metastatic, refractory, or recurrent cervical cancer. Single-agent AXAL was administered at dose levels of 1 × 10^9^, 3.3 × 10^9^, or 1 × 10^10^ colony-forming units (CFU) as an intravenous infusion followed by a second dose 3 weeks later [32]. The investigators reported an acceptable safety profile, with all patients experiencing a flu-like syndrome that responded to symptomatic treatment. At the highest dose, some patients had severe fever and dose-limiting hypotension, but no grade 4 adverse events were reported. Two patients died during the study; the deaths were considered unrelated to the administration of AXAL. Of 13 evaluable patients, five had disease progression, seven had stable disease, and one patient had an unconfirmed partial tumor response with a 32% reduction in tumor load. In a preliminary report of another phase I trial conducted in a similar population of previously treated women with advanced cervical cancer, AXAL was administered at a dose of 5 × 10^9^ or 1 × 10^10^ CFU every 3 weeks for 12 weeks [41]. At the lower dose level, one patient of three experienced grade 3 hypotension as a dose-limiting toxicity. A total of 16 doses were safely administered, and accrual for the second dose level had not started at the time of preparation of this manuscript. Updated data are anticipated.

Two phase II studies of AXAL in women with persistent, recurrent and/or refractory cervical cancer have also been initiated [45, 46]. The first of these evaluates the activity of AXAL in patients with persistent or recurrent cervical cancer, with secondary objectives of evaluating progression-free survival, overall survival, and objective tumor response [45]. Patients will receive AXAL at a dose of 1 × 10^9^ CFU on day 1 with a repeat dose every 28 days for three total doses in the absence of disease progression or unacceptable toxicity. Preliminary data from stage 1 of this trial show that treatment with AXAL led to a 38.5% 12-month overall survival rate in 26 patients. When evaluating safety data, grade 1 or 2 adverse events were reported in 19 of 26 patients (73%), with fatigue, chills, and fever the most common. Only 4 patients (15%) experienced a grade 3 adverse event (e.g., hypotension and cytokine release syndrome) and one patient (4%) experienced a grade 4 adverse event (lung infection and sepsis) [49]. Preliminary data are also available from a second phase II trial of AXAL being conducted in women from India with recurrent/refractory cervical cancer [46]. The primary endpoint of this open-label, randomized phase II study was to determine efficacy and safety of AXAL alone or in combination with cisplatin. In this study, 110 patients were randomized to either one cycle (three doses) of AXAL at 1 × 10^9^ CFU or four doses of AXAL at 1 × 10^9^ CFU together with cisplatin chemotherapy. Following treatment, when analyzing the treatment efficacy in these patients, an 11% response rate was observed, with an average response duration of 10.5 months in both treatment groups. Objective tumor responses included six patients with complete responses and six patients with a partial response; tumor responses were observed in both treatment arms. Another 35 patients had stable disease for more than 3 months, for a disease control rate of 43%. Activity was observed against all high-risk HPV strains detected. The percentage of patients alive at 12 months was 36%, with an 18-month survival rate of 28%. When analyzing treatment safety, two grade 3 serious adverse events were reported, with nonserious adverse events predominantly of transient, noncumulative flu-like symptoms that either spontaneously resolved or responded to symptom-based treatment. The investigators concluded that AXAL can be safely administered in combination with chemotherapy, and is well tolerated with a predictable and manageable safety profile. Moreover, the 36% 12-month survival rate and 11% response rate in this disease setting were encouraging and support the activity of AXAL in recurrent cervical cancer [46].

**Fig. 4:**
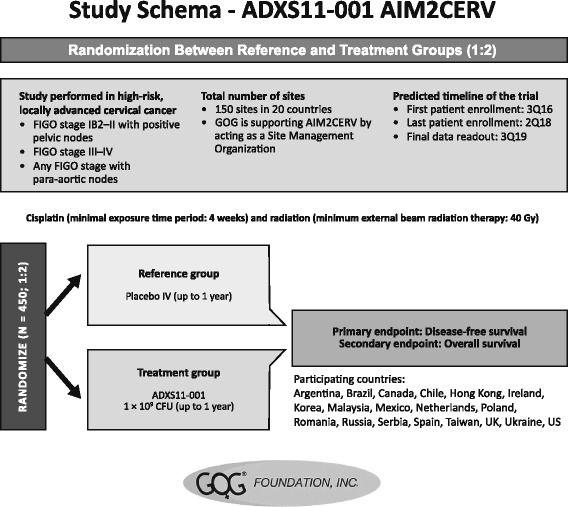
Schematic of the planned AIM2CERV phase III study. FIGO, International Federation of Gynecologic Oncology; GOG, Gynecologic Oncology Group; IV, intravenous; Q, quarter

More recently, a randomized phase III clinical trial (AIM2CERV) enrolling patients with high-risk locally advanced cervical cancer following chemoradiation who will receive AXAL as adjuvant immunotherapy was opened for recruitment in September 2016 [50] (Fig. 4). As patients with high-risk locally advanced cervical cancer present with a 50% probability of recurrence or death following chemoradiation and brachytherapy, there is a clear need for treatment modalities that will lead to improved outcomes. The AIM2CERV trial will evaluate overall survival and disease-free survival of these patients.

The efficacy and safety of AXAL has also been assessed in phase I/II clinical trials that enrolled patients with head and neck cancer, as well as cancer of the anal canal. Although a phase I dose-escalation trial conducted in patients with HPV-16+ oropharyngeal carcinoma was terminated early when two patients suffered dose-limiting toxicities postvaccination [42], a phase I/II trial is currently investigating AXAL and the fully humanized anti–PD-L1 antibody durvalumab alone or in combination in previously treated patients with recurrent/metastatic HPV+ head and neck cancer [44]. The primary objective of the phase I study is to evaluate safety and tolerability of the combination regimen and to select a recommended phase II dose. Preliminary phase I results reported that 10 of the 11 enrolled patients (91%) had treatment-related adverse events, with the majority being grade 1 (7/11; 64%) or 2 (6/11; 55%), such as chills and/or rigors, fever, nausea, hypotension, diarrhea, fatigue, tachycardia, or headache [51]. The primary objective of phase II is to evaluate tumor response, progression-free survival, and safety of AXAL and durvalumab as monotherapy and in combination. A phase II trial in this setting is currently evaluating AXAL in patients with stage II–IV HPV+ oropharyngeal cancer prior to robotic surgery [47]. The primary objective is to determine the immunogenicity of AXAL. Preliminary data have yet to be reported.

In anal cancer, a phase II trial is currently evaluating AXAL as single-agent therapy in patients with persistent/recurrent, locoregional or metastatic anal cancer [48]. Finally, a phase I/II trial is evaluating the combination of AXAL, mitomycin, 5-fluorouracil, and intensity-modulated radiation therapy in patients with anal cancer [43]. The first efficacy and safety data from these trials are expected to be available in 2017.

A topic of interest when evaluating AXAL in the clinical setting is its safety profile, particularly when administered in patients with persistent, recurrent, or metastatic disease who would benefit from co-administration of other immunotherapies. Preliminary evaluation of safety data from phase I/II trials with AXAL, administered alone or combined with other immunotherapeutic agents in patients with HPV+ cervical or head and neck cancers, reported that most adverse events were grade 1 or 2 and included fatigue, chills, fever, and nausea as the most common [46, 51]. Combined administration of AXAL with the anti–PD-L1 antibody durvalumab led to a similar range of adverse events as did AXAL monotherapy. In view of these preliminary results, it can be hypothesized that anticipated toxicities upon combination of AXAL with other immunotherapeutic agents would mainly consist of grade 1–2 adverse events similar to those already reported. Additionally, mild adverse events associated with infusion of AXAL could potentially be observed on the day of dosing; nevertheless, as previously described, these are transient and either self-resolve or respond readily to symptomatic treatment [46].

Another relevant aspect of immunotherapy with AXAL is the identification of predictive and prognostic biomarkers that might be evaluated upon treatment of HPV+ cancer patients, along with expected translational endpoints. In recent years, a relatively wide array of both cellular and molecular biomarkers predictive or prognostic for response to immunotherapy have been identified. Cellular biomarkers relevant for response to AXAL immunotherapy could potentially be of both anti- and protumoral effect. T-cell infiltration of various types of human tumors has been previously reported to be associated with improved clinical outcome [52, 53], whereas high numbers of circulating protumoral immune cell populations, such as MDSCs or Tregs, have been associated with worse overall survival [54, 55]. Considering that AXAL administration results in a decrease in the ratio of Tregs and MDSCs to antitumoral immune effector cells (Fig. 1) [10], these immunosuppressive cell populations might serve as useful prognostic biomarkers for immune response to AXAL. In addition to cellular biomarkers, several molecular biomarkers have been identified as predictors of response to immunotherapy. One notable example is IFN-γ, whose elevated expression in pretreated tumors is associated with clinical response [56]. Other relevant biomarkers for response to AXAL immunotherapy, particularly when administered in combination with immunotherapeutic agents such as durvalumab, are high levels of immune checkpoint molecules such as PD-L1; patients with high PD-L1 expression have been shown to be more likely to benefit from immunotherapy [57]. These cellular and molecular biomarkers could potentially predict response to AXAL, used as monotherapy or in combination with other immunotherapeutic agents, and therefore warrant further investigation.

In view of the often-severe disease burden experienced by cancer patients, acquisition of patient-reported outcomes, along with response to treatment, would help provide comprehensive clinical insights. Systematic measurements of these patient-reported outcomes are possible today with the use of existing validated tools. Two of the most commonly used measurement systems are the Functional Assessment of Anorexia/Cachexia Therapy (FAACT) and the Functional Assessment of Chronic Illness Therapy – Fatigue (FACIT-F) questionnaires, developed for assessment of anorexia/cachexia and fatigue experienced by cancer patients undergoing various treatments [58, 59]. Taking into consideration the preponderance of fatigue and nausea associated with the adverse events observed to date in clinical trials with AXAL immunotherapy, the patient-reported outcomes mentioned above bear relevance and could potentially be investigated in future clinical trials of AXAL.

## Conclusions

*Lm*-based immunotherapy has progressed considerably since the completion of the first preclinical studies. Genetic engineering, utilized to obtain a recombinant, attenuated form of *Lm* as a bacterial vector, has enhanced the safety of *Lm*-based vaccines such that they have now been utilized successfully in clinical trials in humans. Moreover, the fusion of tumor antigens to LLO has greatly enhanced the immunologic and antitumor properties of these vaccines. As several studies have indicated, one major challenge for *Lm*-based vaccines is their capacity to induce CD25+ Treg cells with a propensity for immunosuppression along with the CD4+ and CD8+ effector T cells that are needed for protective immunity [28, 35]. However, this effect can be overcome by combining *Lm*-based vaccines with other targeted antitumoral therapies, such as monoclonal antibodies [40]. The current clinical status of AXAL, which continues to be assessed in patients with HPV-associated cancers at different stages, provides optimism for the future of the vaccine in the treatment of these malignancies. Administered alone or in combination with various cancer therapies, AXAL has been proven to be well tolerated by patients with HPV-associated cancers in multiple investigations, with early promising signs of antitumor activity also being reported. These encouraging findings pave the way for AXAL phase III clinical trials and, at later stages, the potential introduction of AXAL into the clinical setting.

## Abbreviations

ActA: Actin nucleator A
APC: Antigen-presenting cell
AXAL: Axalimogene filolisbac or ADXS11-001
CD: Cluster of differentiation
CFU: Colony-forming unit
CTL: Cytotoxic T lymphocyte
CTRI: Clinical Trials Registry – India
CXCL9: Chemokine (C-X-C motif) ligand 9
DC: Dendritic cell
FAACT: Functional Assessment of Anorexia/Cachexia Therapy
FACIT-F: Functional Assessment of Chronic Illness Therapy – Fatigue
FIGO: International Federation of Gynecologic Oncology
GOG: Gynecologic Oncology Group
HER2: Human epidermal growth factor receptor 2
HPV: Human papillomavirus
IFN-γ: Interferon gamma
IL: Interleukin
IMRT: Intensity-modulated radiation therapy
IV: Intravenous
LLO: Listeriolysin O
*Lm*: *Listeria monocytogenes*
MDSC: Myeloid-derived suppressor cell
MHC: Major histocompatibility complex
NCT: National Clinical Trial
NLR: Nucleotide-binding oligomerization domain-like receptor
PD-1: Programmed cell death protein 1
PD-L1: Programmed cell death protein 1 ligand 1
PD-L2: Programmed cell death protein 1 ligand 2
prfA: Protein-related factor A
Q: Quarter
TAA: Tumor-associated antigen
tLLO: Truncated listeriolysin O
TLR: Toll-like receptor
TME: Tumor microenvironment
Treg: Regulatory T cell
